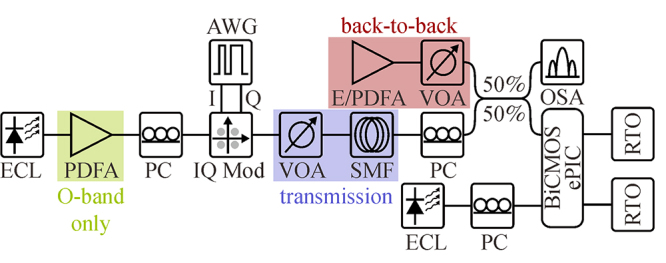# Toward coherent O-band data center interconnects

**DOI:** 10.1007/s12200-021-1242-0

**Published:** 2021-10-27

**Authors:** Pascal M. Seiler, Galina Georgieva, Georg Winzer, Anna Peczek, Karsten Voigt, Stefan Lischke, Adel Fatemi, Lars Zimmermann

**Affiliations:** 1 Technische Universität Berlin, Chair of Siliziumphotonik, Berlin, 10623 Germany; 2 IHP - Leibniz-Institut für innovative Mikroelektronik, Frankfurt (Oder), 15236 Germany; 3 Technische Universität Berlin, Chair of Hochfrequenztechnik-Photonik, Berlin, 10623 Germany; 4 IHP Solutions GmbH, Frankfurt (Oder), 15236 Germany

**Keywords:** coherent communication, data center, O-band, silicon photonics

## Abstract

Upcoming generations of coherent intra/inter data center interconnects currently lack a clear path toward a reduction of cost and power consumption, which are the driving factors for these data links. In this work, the tradeoffs associated with a transition from coherent C-band to O-band silicon photonics are addressed and evaluated. The discussion includes the fundamental components of coherent data links, namely the optical components, fiber link and transceivers. As a major component of these links, a monolithic silicon photonic BiCMOS O-band coherent receiver is evaluated for its potential performance and compared to an analogous C-band device.

## 1 Introduction

Coherent communication is emerging in the intra data center domain to support the growth of traffic. While the advantages of deploying coherent communication for shorter link distances is apparent, there is as yet no clear path to reduce the cost that is typically associated with coherent long-haul communication. Since cost is the major driving factor for data center interconnects (DCIs), there is an obvious need for cost reducing solutions to future coherent DCI communication links. The continued use of C-band coherent photonics for intra/inter DCIs may seem like a natural step, as C-band coherent links are the only feasible option in long-haul communication. However, given their inherent susceptibility to chromatic dispersion (CD) and the disadvantages of compensating filters, an evaluation of alternative approaches is needed. An alternative is the deployment of O-band coherent links, due to their zero-dispersion window around 1310 nm. The O-band is already the present standard for intra DCIs. However, achieving data rates beyond 1.6 Tbps will be challenging for direct detection-based systems [1]. The potential deployment of coherent links in comparison to direct-detection based systems has sparked an intense discussion [1–5]. However, the discussion regarding the deployment of O-band coherent DCIs has been limited [6,7]. As of yet, there has not been a comparative study aiming to investigate the trade-offs and advantages of the use of O-band coherent photonics in comparison to C-band for data center deployment.

This work pursues this topic with a detailed discussion, addressing each aspect of a coherent DCI. The targeted link distances investigated in this work ($\leqslant$20 km) are deliberately chosen to be outside the typical intra DCI length (approx. 2 km), since an optimal transition point for C- to O-band coherent photonics is not necessarily indicated by the present knowledge.

The structure of this paper is as follows: Sections 2.1 to 2.3 address wavelength dependencies in the performance of on-chip components, namely waveguide loss, fiber to chip couplers, and 90° hybrids. Sections 2.4 and 2.5 discuss trade-offs due to the fiber link and potentially reduced digital signal processing (DSP). Finally, Sections 2.6 and 2.7 compare the performance of O- and C-band transmitters and receivers. A silicon photonic coherent BiCMOS O-band receiver [8] is compared to an analogous C-band device fabricated in the same technology. As one of the major components in a DCI, the O-band receiver is evaluated via a transmission experiment at 56 GBd. Finally, a conclusion is given in Section 3.

## 2 Silicon photonic components for coherent DCIs

For a cost driven market like that for DCIs, a highly

Topical Collection: Recent Advances in Silicon Photonics scalable base technology is required to reduce the cost. For that reason, silicon photonics has gained a lot of momentum in recent years. In addition, it has been proven that silicon photonics can efficiently co-integrate photonics and electronics for high-speed transceivers. For this work, exemplary devices have been manufactured in IHPs 0.25 µm photonic BiCMOS technology, which monolithically co-integrates bulk-Si high-speed radio frequency (RF) frontend electronics and high-speed, 220 nm silicon-on-insulator (SOI) photonic devices [9,10].

## 2.1 Waveguide loss

The propagation loss in waveguides can be mainly differentiated in: 1) linear losses and 2) higher-order non linearities like two-photon absorption (TPA) and the associated increased free carrier absorption (FCA) [11,12].

1) Linear loss

The linear losses depend on the specific waveguide geometry [11]. Major factors are manufacturing related non-idealities. Among these are, e.g., scattering due to surface roughness, increased bend loss due to sidewall angles [13], or thickness variations. These are difficult to separate, and in practice, a single loss value is usually obtained. Exemplary values for the linear loss have been determined with a schematic of the waveguide geometry shown in Fig. 1(a). The waveguide structures are designed as rib waveguides with 220 nm silicon (Si) on 2 µm $SiO_2$. The etch depth $d$ is 70 nm. For the C-band waveguides, a core width $w$ = 700 nm and slab width of 3.65 µm is used. For the O-band, this is changed to $w$ = 590 nm and 2 µm slab width. Average linear loss values of fully integrated waveguides for the C- and O-band of 0.92 and 0.87 dB/cm respectively have been determined using optical backscattering reflectometry at wafer scale [14]. A wafer distribution of the linear loss in the O-band is given in Fig. 1(b).

2) TPA

Direct-detection based links require a sufficiently high laser power to be launched into the transmitter to support reach and symbol rate requirements. In future coherent DCIs, this will also be accompanied with a strong local oscillator to support increasing symbol rates and thus

**Fig. 1** (a) Waveguide geometry of a rib waveguide in 220 nm thick silicon-on-insulator (SOI) on a 2 µm $SiO_2$ box. (b) Wafer distribution of the linear loss in the O-band for a rib waveguide. The black markers indicate invalid measurements. BEOL: back end of line. The red dashed box indicates the area for the nonlinear effective area calculation higher receiver sensitivities [5,15]. While this affects both O- and C-band, the O-band potentially suffers from increased nonlinear losses within the waveguide. These are governed by TPA and the associated generation of carriers leading to FCA, in which two photons are absorbed for the generation of one phonon and an electron-hole pair. The optical power absorbed along a waveguide longitudinal $z$-direction due to TPA, $P_{\mathrm{TPA}}$, may be expressed as [12]

$$P_{\mathrm{TPA}}(z) = \frac{\beta_{\mathrm{TPA}}}{A_{\mathrm{nl,eff}}} \cdot \int_0^z P^2(z)\mathrm{d}z, \tag{1}$$

where $\beta_{\mathrm{TPA}}$ is the TPA coefficient, and $P$ is the optical power. The effective area used in Ref. [12] is in this work replaced with a nonlinear effective area $A_{\mathrm{nl,eff}}$ based on Ref. [16], due to the negligible attribution of the waveguide cladding to the nonlinear interaction in SOI waveguides. Omitting the marginal difference in linear losses, the power within the waveguides is the same for both O- and C-band operation. The difference in loss due to TPA is thus largely impacted by the prefactor in Eq. (1). In applications where a strong nonlinear interaction is desirable, like four-wave-mixing [16], the nonlinear effective area is minimized. However, in transceivers this is highly undesirable, and the waveguides are often designed for a minimal waveguide loss. An estimation for the difference in TPA between O- and C-band for transceivers is provided hereinafter. First, the nonlinear effective areas are determined by simulating the waveguide modes using FIMMWAVE (Photon Design) and calculation of the nonlinear effective area is based on Ref. [16]. While the derived nonlinear effective area in Ref. [16] assumes strip- or slot waveguides, the waveguides under investigation in this work are rib waveguides with a shallow etch (70 nm). To accommodate for that, rectangular waveguides with a 200 nm wider core width than the actual width are assumed to include the part of the mode that is guided in the slab area. The area considered for the nonlinear interaction is also shown as a red dashed box in Fig. 1(a). With the waveguide dimensions from the previous section, the nonlinear effective areas for the fundamental transverse electric (TE) mode at 1310 and 1550 nm are 0.14 and 0.18 µm$^2$, respectively. An estimation for $\beta_{\mathrm{TPA}}$ at 1310 nm can be based on the wavelength dependence of the TPA coefficient, which can be expressed as [17]

$$\beta_{\mathrm{TPA}}(\lambda) = C_2 \cdot F_2^{\mathrm{ind}}(\lambda), \tag{2}$$

where $C_2$ is an only weakly varying material parameter, $\lambda$ is the wavelength, and $F_2^{\mathrm{ind}}(\lambda)$ is a dimensionless function dependent on the wavelength, of the form [17]

$$F_2^{\mathrm{ind}}(\lambda) = \frac{\left(2 \cdot \dfrac{hc/\lambda}{E_{\mathrm{ig}}} - 1\right)^4}{\left(2 \cdot \dfrac{hc/\lambda}{E_{\mathrm{ig}}}\right)^7}. \tag{3}$$

Here, $h$ is the Planck constant, $c$ is the speed of light, and $E_{ig} \approx 1.12$ eV is the indirect bandgap of silicon. A reported value for $\beta_{TPA}$ in this technology is 0.56 cm/GW at 1550 nm [18]. With Eq. (3) solved for 1550 nm, material parameter $C_2$ is determined from Eq. (2) to be approximately 2.01 $\times 10^2$ cm/GW. Then, with $F_2^{ind}$ solved for 1310 nm, it follows that the TPA coefficient $\beta_{TPA}$ at 1310 nm is approximately 1.16 cm/GW. Neglecting the marginal difference in linear losses, the change in absorption due to TPA between 1310 and 1550 nm is then calculated based on Eq. (1) as

$$\frac{P_{TPA,\,1310\,nm}}{P_{TPA,\,1550\,nm}} \approx \frac{\left.\frac{\beta_{TPA}}{A_{nl,eff}}\right|_{\lambda=1310\ nm}}{\left.\frac{\beta_{TPA}}{A_{nl,eff}}\right|_{\lambda=1550\ nm}} = \frac{8.29\frac{cm}{\mu m^2 \cdot GW}}{3.11\frac{cm}{\mu m^2 \cdot GW}} \approx 4.3\ dB. \tag{4}$$

Assuming a saturation power of +25 dBm at 1550 nm [18], the penalty estimated here, due to the increase in TPA, indicates an onset of the saturation at roughly 20.7 dBm. In approximation, this would still allow for optical powers within the waveguide of about +20 dBm, which would suffice for most practical applications.

### 2.2 In-/out-coupling

For DCI applications, a low-cost fiber coupling manufactured in a scalable technology is required. For that purpose, grating couplers (GRCs) are already established for intra DCIs [19,20]. For dual polarization applications, 1D-GRC (single polarization) are extended toward 2D-GRC (polarization independent), which are of a particular interest due to their intrinsic polarization splitting and the possibility of on-wafer testing. Presently, coupling efficiencies for 2D-GRC in different silicon photonics platforms between −3.2 and −3.8 dB have been reported [21,22]. Further improvements to the coupling efficiency by approximately 1 dB may be achieved by the integration of back-side metallization [23], or a double-SOI substrate [24]. Other available options for chip coupling using a standard single mode fiber (SMF) include spot size converters by means of inverse tapers [25], multi-rod structures [26], subwavelength gratings [27], or polymer interfaces [28]. Typically reported coupling efficiencies are between 1 and 2 dB. However, on-wafer testing using edge-coupled approaches is not yet established. While potentially more power efficient, they also come with the disadvantage of increased fabrication challenges during the co-packaging [28], specifically mechanical stability and epoxy "shrinkage" during the curing process, which can displace the fiber array [29]. Considering the compatibility of GRC with large-scale manufacturing [29] and the availability of wafer-level testing, GRC are the most mature option for mass market entry.

To directly compare the implementation of O- and C-band 2D-GRC in the same technology, we have fabricated respective pairs of 1D- and 2D-GRC, designed for either O-band or C-band operation. The devices are fabricated using a 248 nm deep UV lithography without a back end of line (BEOL) process. The BEOL stack increases the coupling distance, which causes additional losses [30]. The fabricated devices are two directly connected 1D-GRC and a 2D-GRC with 1D-GRC at each output and schematics are shown in Fig. 2(c). Ten structures distributed over the wafer are measured for a statistical analysis. Exemplary results for the coupling efficiency per coupler for the C- and O-band are shown in Figs. 2(a) and 2(b), respectively. An accurate comparison of the 1D- and 2D-GRCs is only possible for one polarization. For that reason, we use the 1D-GRC as input and measure the transmission at the 2D-GRC output. The waveguide losses are negligible. The coupling efficiencies (mean±standard deviation) for the O- and C-band 2D-GRC are (3.7±0.2) and (4.4±0.2) dB, respectively. Only the transmission for one of the 1D-GRC inputs is shown due to the devices symmetry. It is evident that a similar performance for O- and C-band 2D-GRC may be achieved, while using a highly scalable technology supporting co-integrated electronics and a 248 nm deep UV lithography. Note that the slightly improved performance of the O-band gratings is not a fundamental property, but rather technology dependent. However, a full optimization of either wavelength requires an additional effort, and state of the art performance in both bands simultaneously is difficult to achieve.

### 2.3 90° hybrid

Early development of coherent receivers in the C-band focused on 90° hybrids, either using networks based on 2 × 2 multi-mode interference (MMI) couplers [31], or a single 4 × 4 MMI [32]. Since then, C-band optical 90° hybrids have been researched in detail [33–35]. A 90° hybrid based on a 4 × 4 MMI is designed with 1310 nm central wavelength and a schematic with port enumeration is shown in Fig. 3(c). The MMIs core width is 10 μm and its length is 238.5 μm. Each port is connected with a 25 μm long taper for waveguides with a core width of 600 nm and a slab width of 2.5 μm (comp. Fig. 1(a)). The simulated 1 dB loss bandwidth is approximately 50 nm. Simulation results for the differential imbalance and the normalized phase error are given in Figs. 3(a) and 3(b), respectively. The dashed lines indicate the performance under assumption of typical process variations in this technology [36]. In Fig. 3(a), the imbalance is given as the differential error when using either port 1 or 3 as optical input. In Fig. 3(b), the phase error is given relative to output port 1. The

**Fig. 2** Coupling efficiencies for (a) C-band, (b) O-band 1D and 2D grating couplers (GRCs). The structures have been fabricated without a BEOL process. (c) Schematics of the measured structures

**Fig. 3** (a) Differential multi-mode interference (MMI) imbalance. (b) MMI phase error relative to output 1. The dashed lines in (a) and (b) account for typical fabrication tolerances. (c) Schematic of the MMI. i: input; o: output imbalance and phase error remain over a bandwidth of 40 nm (which is chosen to be comparable to the grating couplers) in both scenarios below 1 dB and 1.7°, respectively. This is comparable to analogous C-band devices in the same technology [32].

## 2.4 Standard fiber link (SMF28)

One of the major points considering the fiber link is the difference in fiber loss between O- and C-band. The increase in fiber attenuation in the O-band compared to C-band in a widely used fiber is for example 0.14 dB/km [37]. Over 10 km, this amounts to an additional loss of 1.4 dB in the O-band. Optical amplification is infeasible for both O- and C-band operation within data centers, due to constrains on the power consumption. A compensation for this additional penalty could be achieved by an increased power at the local oscillator, which would increase the receiver sensitivity [15]. While also applicable in the C-band, justification for this additional expense for operation in the O-band may be found in the second point considering the fiber link. The chromatic dispersion exhibits a zero dispersion point between 1300 and 1324 nm [38]. This makes dedicated CD compensation for communication within that window negligible, and can lead to an overall power efficiency improvement, which is to be discussed in further detail in the next section. While special fibers (like dispersion compensating fibers and dispersion shifted fiber) to address the CD in the C-band exist and are being deployed in long-haul communication, they add cost and complexity to the system. Given the strict boundary condition in the intra-/inter data center domain being cost, these should be avoided.

### 2.5 DSP

The DSP for long-haul C-band coherent data links consists on the transmitter side chiefly of digital-to-analog converter (DAC) and pulse shaping. On the receive side, the functional blocks are analog-to-digital converter (ADC), carrier- and clock recovery, forward error correction (FEC), and compensating filters for chromatic dispersion and polarization mode dispersion (PMD). While most of these operations are still required at shorter link distances, PMD may be ignored at sufficiently short link distances [6], independent of the wavelength. The compensation for CD may also be neglected, depending on the wavelength, as is discussed hereinafter.

The general issue concerning CD is a broadening of the modulated pulse, which leads to inter symbol interference (ISI). This may cause two or more symbols to overlap, and hence impede the symbol interpretation. Intuitively, this impact grows with increasing accumulated CD, i.e., by longer fiber links. Additionally, higher symbol rates, having shorter symbol durations, are more affected by accumulated CD than lower symbol rates. Whereas roughly 30 GBd is a common symbol rate for present DCIs, future generations of DCIs, supporting 800 Gbps and beyond, would likely operate at $>64$ GBd [5]. In principle, DSP-based coherent transceivers in the C-band can mitigate all of the accumulated CD, independent of the symbol rate, by deploying a suitable digital filter [15]. However, this comes at the expense of increased power consumption. Presently, the compensation for CD, PMD, and the polarization demultiplexing account for more than half of the receiver's power consumption [15]. To be mass market viable, future coherent DCIs therefore need approaches to reduce power consumption. While for the C-band, a digital filter needs to be deployed, the DSP for compensating the CD can be completely omitted by deploying a coherent data link in the zero-dispersion window (1300–1324 nm). However, given the large spectral width of the O-band (1260 to 1360 nm), CD compensation at the edges of the band might become relevant again. An estimation of the required filter complexity and resulting power consumption is performed in the remainder of this section. For the simulation, wavelengths of 1260, 1310, and 1550 nm are investigated. A list of the simulation parameters including measured fiber dispersion coefficients for 1260 and 1310 nm are given in Table 1.

First, the number of taps $N_{\mathrm{CD}}$ for a CD compensating filters may be calculated as [39]

$$N_{\mathrm{CD}} = \left\lceil n_{\mathrm{os}} \cdot \frac{\lambda^2}{c} \cdot |l \cdot D| \cdot R_{\mathrm{S}}^2 \right\rceil. \tag{5}$$

Here, $n_{\mathrm{os}}$ is an oversampling factor, $l$ is the length of the fiber link, $D$ is the fibers dispersion coefficient at wavelength $\lambda$, $R_{\mathrm{S}}$ is the symbol rate and $c$ is the speed of light. A dual polarization quadrature amplitude modulation 16 (DP-QAM-16) link at 20 km within a 7 nm CMOS technology process is assumed. The symbol rate and wavelength are varied and the resulting filter complexity is calculated based on Eq. (5). Only one link distance is investigated due to the dominance of the symbol rate in Eq. (5). Based on the number of filter taps, the number of complex multiplications per information bit $C_{\mathrm{CD}}$ is then [39]

$$C_{\mathrm{CD}} = \frac{N_{\mathrm{CD}} \cdot n_{\mathrm{os}}}{2\log_2 M}, \tag{6}$$

where $M$ is the number of symbols in the symbol alphabet. Assuming a realization of a single complex multiplication by three real multiplications and five real additions, the energy per complex multiplication is then calculated using the energy models presented in Ref. [40]. For this, a CMOS feature size of 7 nm and supply voltage of 0.8 V is considered [40]. The results for the filter complexity and power consumption are shown in Figs. 4(a) and 4(b), respectively. Due to the quadratic dependency in Eq. (5), the filter complexity for a 1550 nm link rapidly increases with the symbol rate and a 60 GBd link would require approximately 13 taps at a power consumption of roughly 1.6 W. Assuming a worst-case O-band link at 1260 nm, the power consumption at 60 GBd would still be below 0.4 W. The filter complexity is also simulated for a link at 1310 nm. However, the number of taps remains at 1 up at 90 GBd, which is the theoretical minimum in Eq. (5). The power consumption thus also remains negligibly small throughout the simulation. Finally, the simulation results is compared to a reported coherent 400G ZR module from Inphi Corp., supporting a large spectrum of link distances, including DCIs down to link distances of 2 km. The module also deploys single lane DP-QAM-16 at 60 GBd in a 7 nm CMOS technology [41]. The reported power dissipation is 4 W/100 Gbps. Here, we assume that the DSP accounts for approx. 50% of the total power consumption [41], and that the CD compensation accounts for approx. 25% of the DSP power consumption [40]. Based on the simulated 1.6 W at 60 GBd and 1550 nm, this results in roughly 3.2 W/100 Gbps, which is comparable to the reported module by Inphi Corp.

While this is only a coarse estimate, it is evident that future coherent DCIs will have to maintain a low power consumption to be viable for the mass market, especially at increasing symbol rates. At sufficiently low link distances,

(a) (b)

**Fig. 4** Simulated filter (a) complexity and (b) power consumption of CD compensation for different symbol rates and wavelengths at a targeted link distance of 20 km. Only one link distance is investigated due to the symbol rate being the dominant factor in the filter complexity

**Table 1** CD compensation simulation parameters

| variable | | value | |
|---|---|---|---|
| modulation format | | DP-QAM-16 | |
| nominal ADC resolution $n_{adc}$ | | 4 [43] | |
| DSP resolution | | $n_{adc} + 2$ [40] | |
| oversampling factor $n_{os}$ | | 1.25 [43] | |
| fiber length | | 20 km | |
| wavelength $\lambda$ | 1260 nm | 1310 nm | 1550 nm |
| fiber dispersion coefficient $D$ | $-5$ ps/(nm·km) [a)] | $-0.7$ ps/(nm·km) [a)] | 17 ps/(nm·km) |
| CMOS process technology feature size | | 7 nm | |
| CMOS supply voltage | | 0.8 V [40] | |

**Note**: a) Measured values using a standard SMF

PMD may also be neglected, further decreasing power consumption. Optimal solutions require a data center specific CMOS-related trade-off between digital filter complexity and power consumption. Noteworthy potential candidates for these are DSP-free links [7,42]. However, due to the absence of DSP, they are inherently more susceptible to CD than DSP-based links, and only a few kilometers of C-band transmission can have a significant impact [7], which further supports O-band coherent DCIs.

## 2.6 Transmitter

The most commonly utilized effect for silicon photonic high-speed inphase/quadrature (IQ) modulators is the plasma-dispersion effect, in which a change in the free carrier concentration leads to a change in the refractive index [44]. While the efficiency of this refractive index change is lower for the O-band than for the C-band ($\approx$ 70%), this is offset by a reduced increase in absorption (also $\approx$ 70%) and overall shorter phase shifter length in the O-band [45]. The development of high-speed silicon photonic IQ modulators has been ongoing for many years [46–49]. Recent works have demonstrated up to 100 GBd QAM-32 using a silicon photonic modulator with a 3-dB bandwidth of $\approx$ 34 GHz [50]. However, this requires a driving voltage of $\approx$ 5 $V_{PP}$, which is difficult to implement in a low power environment. At a reduced power consumption, 100 GBd quadrature phase shift keying (QPSK) and 50 GBd QAM-16 has been shown with driving voltages of $\approx 3.2V_{PP}$ and $\approx 1.6V_{PP}$, respectively [51]. The investigation of silicon-based IQ modulators in the O-band is presently still limited [52,53], but 77 GBd QPSK with a driving voltage of $\approx 3V_{PP}$ and 45 GBd QAM-16 have been displayed [53]. In the technology used in this work, 64 GBd DP-bipolar-8ASK has been demonstrated in the C-band, which could be extended toward higher order modulation formats [54]. It is evident that further advances regarding the power efficiency of silicon modulators are required to meet the challenging demands in DCs, which is an ongoing process [55]. Additionally, the co-integration of other electro-optic materials on silicon is gaining attention. While still an active development and research topic that will not mature in the near future, it might offer an alternative approach to 100 GBd interconnects [56] in a low cost and power consumption environment.

### 2.7 Receiver

There has been extensive research on the fabrication of monolithic silicon-based coherent receivers at 1550 nm [57–64], with recent works demonstrating a transmission at 64 GBd using a device fabricated in the same technology as used in this work [64]. Since technology specifications, design parameters and equipment can vary, a direct comparison of O- and C-band performance for a receiver is quite difficult. For this purpose, we have fabricated two coherent receivers with the same electrical topology [65], optimized for either O-band [8] or C-band. The optical loss contributions of both the O- and C-band receivers are compared in Table 2. The insertion loss for the GRCs and waveguide routing are determined using process control structures. The MMIs for both O- and C-band have been simulated, and for the O-band the MMI in Section 2.3 has been used. Due to the phase relation of 4 × 4 MMIs, waveguide crossings have to be used and their performance is also simulated. Each loss contribution is listed in Table 2 together with the respective values in a 40 nm bandwidth. The expected loss for the optical circuit at 1310 nm accumulates to approximately 5 dB and deviates less than 0.2 dB compared to that of the C-band device, which is negligibly small. That difference remains below 0.5 dB over a 40 nm bandwidth. To compare the performance of both receivers, an intradyne back-to-back transmission has been performed. A detailed description of the deployed setup in Fig. 5(a) can be found in Ref. [8]. It is noteworthy that the IQ modulator is a dedicated C-band device and is used to evaluate both receivers, as commercial O-band modulators are as yet not readily available. The praseodymium-doped fiber amplifier (PDFA) is used during the O-band measurement to allow for similar power levels at the receiver input for the O- and C-band measurement. The O-band laser is amplified to +16 dBm, which is within the available fiber coupled optical power for reported devices at that wavelength [66]. To compare the two coherent receivers, bit error rate (BER) measurements are performed at varied optical signal to noise ratios (OSNRs) using either a PDFA or erbium-doped fiber amplifier (EDFA) for noise loading. Figure 5(b) shows the BER over the OSNR (in 0.1 nm in the C-band, and 71.6 pm in the O-band) for a 56 GBd QPSK using either the O-band or C-band receiver in a back-to-back scenario. For the O-band receiver, 48 GBd QPSK is also shown for reference. While amplifier-free

**Fig. 5** (a) Intradyne setup for back-to-back and transmission experiment. A local oscillator power of ≈ +12 dBm has been maintained. (b) Bit error rate (BER) vs. optical signal to noise ratio (OSNR) for measured 56 GBd QPSK (B2B) using a dedicated O- and C-band receiver and in theory. Also shown is the BER vs. OSNR for 48 GBd QPSK using the O-band receiver and in theory. (c) BER vs. received optical power for 56 GBd QPSK at different link distances using an O-band receiver. The margins indicate the total remaining power budget. ECL: external cavity laser; PC: polarization controller; IQ Mod: IQ modulator; AWG: arbitrary waveform generator; VOA: variable optical attenuator; SMF: single mode fiber; OSA: optical spectrum analyzer; RTO: real time oscilloscope

**Table 2** O- and C-band loss per component

| component | loss/dB | | | |
|---|---|---|---|---|
| | 1310 nm | (1310±20) nm | 1550 nm | (1550±20) nm |
| grating coupler | 4.1[a)] | < 6.7[a)] | 4.3 | < 7.2 |
| waveguide | | 0.2 | | |
| MMI | 0.3 | < 1 | 0.3 | < 1 |
| crossing | | 0.4 | | |
| total | 5 | < 8.3 | 5.2 | < 8.8 |

**Note**: a) Measured at (1290±20) nm, due to the coupling angle links are not limited by the OSNR, but rather the received optical power, OSNR measurements are the typical method by which coherent receivers are evaluated and compared. Also shown in Fig. 5(b) is the received optical power for the modulated signal for O- and C-band, respectively. The performance is, as expected, quite similar, with a slightly decreasing performance in the O-band for increasing OSNRs. Since the electrical topology and expected bandwidth is identical for both receivers, this can be reasonably assumed to be largely attributed to the added imbalance and phase error of the C-band modulator during the O-band measurement. It should be noted, that the remaining singular bit errors at high OSNRs (e.g., 30 dB) are potentially setup-related. Consecutive blocks have been recorded and processed until enough received bits become available to support the statistical analysis. The relatively long measurement time per error rate (up to $\approx 30$ min for high OSNRs), and the unavoidable instability of the coupling in that time affects the BER measurement, and may cause individual errors. The O-band signal has also been pre-amplified, which further degrades the performance slightly (PDFA noise figure $< 7$ dB). Using the O-band receiver, a transmission experiment through standard SMF (average loss in the O-band 0.46 dB/km) has also been performed. The optical received power is varied using a variable optical attenuator (VOA). No compensation for CD is added to the DSP. The minimal received optical power is $\approx -20$ dBm. The results in Fig. 5(c) show power margins of 7, 5.3, and 3.8 dB for the investigated link distances of 4, 8.5, and 13 km, respectively.

## 3 Conclusions

Within this work, the O- and C-band performance of all major components required for a silicon-based coherent DCI were compared in a photonic BiCMOS platform. The significant wavelength dependencies on the performance were found in the nonlinear loss due to TPA, the fiber link, and DSP. While increased nonlinear losses in the O-band lead to a penalty for the achievable power within the waveguide, this still allows for $\approx +20$ dBm. For practical applications, this should not be a limiting factor, as signals are split on either end of a transceiver. In the receivers demonstrated here, the waveguide length until the 90° hybrid is approximately 1.1 mm, which further limits the impact of TPA. The deployment of O-band coherent photonics for DCIs thus greatly depends on a trade-off between fiber propagation loss and power savings due to reduced DSP. Within the fiber link, the performance of O-band communication suffers due to increased losses, though the penalty of this increased O-band loss becomes significant only for distances of several kilometer. On the other hand, for a C-band coherent link a CD compensating filter is required. Given the strict boundaries being cost and power efficiency, it is evident that an overall reduced DSP complexity paired with the O-band can mitigate issues faced by future coherent DCIs. Meeting these requirements is essential for a large-scale deployment. Nonetheless, further concerns, like the maturity of coherent C-band photonics, need to be addressed for a potential application. Additional efforts will have to be made to develop the photonic transceiver structures, e.g., the 90° hybrid on the receive side. However, O-band laser and 2D-GRC are already well established in the data center domain, reducing that expense significantly. A major original driving factor for C-band coherent photonics was the establishment of C-band optical amplifiers. While O-band amplifiers are presently inferior in terms of the output power and gain, amplification is generally undesirable in data centers. A paradigm shift from C- to O-band coherent photonics for data centers is thus favorable in our opinion, and a promising candidate for satisfaction of power consumption demands, especially at link distances of just a few kilometers [6,67]. The advantages offered by using the O-band, which is the present and near future standard in data centers, also support coherent communication and more so for increasing symbol rates. Future work shall continue to investigate the specific dependencies on link distance, DSP filter complexity, and power consumption for a beneficial shift to O-band coherent links within data centers.

**Acknowledgements** This work was supported in part by the German Research Foundation (DFG) through the projects EPIC-Sense (ZI 1283-6-1) and EPIDAC (ZI 1283-7-1), by the Federal Ministry of Education and Research (BMBF) through project PEARLS (13N14932), and the European Commission through project H2020-SPACE-ORIONAS (822002).
